# PTSD correlates with somatization in sexually abused children: Type of abuse moderates the effect of PTSD on somatization

**DOI:** 10.1371/journal.pone.0199138

**Published:** 2018-06-21

**Authors:** Seung Min Bae, Jae Myeong Kang, Hyoung Yoon Chang, Woori Han, So Hee Lee

**Affiliations:** 1 Department of Psychiatry, Gil Medical Center, Gachon University College of Medicine, Incheon, Republic of Korea; 2 Sunflower Children’s Center, Incheon, Republic of Korea; 3 Department of Psychiatry and Behavioral Sciences, Ajou University School of Medicine, Suwon, Republic of Korea; 4 Department of Psychiatry, National Medical Center, Seoul, Republic of Korea; Stellenbosch University, SOUTH AFRICA

## Abstract

**Purpose:**

Somatization is a major post-traumatic symptom in sexually abused children. Thus, the present study aimed to determine the relationship between post-traumatic stress disorder (PTSD) symptoms and somatization, and between intelligence and somatization in child sexual abuse victims and to elucidate whether type of abuse had an effect on the relationship between PTSD symptoms and somatization.

**Methods:**

This study evaluated the somatizations (Child Behavioral Checklist/6–18 [CBCL]), PTSD symptoms (Trauma Symptom Checklist for Children [TSCC]), and intelligence levels of 63 sexually abused children. Correlation and regression analyses were performed to predict somatization based on PTSD symptoms, intelligence, age, and type of sexual abuse, and to find moderating effect of type of abuse on the effect of PTSD symptom on somatization.

**Results:**

PTSD symptoms (β = 0.471, *p* = 0.001) and intelligence (β = 0.327, *p* = 0.021) were associated with somatization. Type of abuse was not, by itself, correlated with somatization (β = 0.158, *p* = 0.281), but it did have a moderating effect on the effect of PTSD symptoms on somatization (Type of abuse*PTSD symptoms, β = -0.299, *p* = 0.047). PTSD symptoms were associated with somatization only among those who experienced the molestation type of abuse.

**Conclusions:**

Somatization in sexually abused children was influenced by the severity of PTSD symptoms and intelligence, and the effect of the PTSD symptoms on somatization was moderated by type of abuse. Specifically, the rape type of abuse may attenuate the effect of post-traumatic symptoms on somatization.

## Introduction

Child sexual abuse is a widespread and major public health problem [1]. The definition of sexual abuse ranges from no contact (exhibitionism) to sexual intercourse, and victims can experience a variety of physical and emotional consequences. For example, psychiatric disorders such as major depressive disorder, generalized anxiety disorder, panic disorder, phobias, substance abuse, eating disorders, somatic symptom disorder, and post-traumatic stress disorder (PTSD) are correlated with child sexual abuse [2]. PTSD is one of the most common sequelae of child sexual abuse [3]. Among those who have experienced trauma, sexual abuse victims are the most vulnerable to the development of PTSD; indeed, 48% of this group develop PTSD as adolescents, whereas only 3% of those experiencing natural/other disasters meet criteria for PTSD [4]. Somatizations often follow child sexual abuse [5]. Specific and functional somatizations without a medical cause, such as chronic pelvic pain [6], irritable bowel syndrome, non-epileptic attacks [7], and back pain [8] have been reported to be associated with sexual abuse. Somatization is important in regarding that it can be an adaptive response to psychological distress [9] and accidental disclosure by post-traumatic somatization can initiate the intervention process and minimize the damage of the sexual abuse.

The relationship between PTSD and somatization was demonstrated in several studies. The excess of somatization was found to associate with PTSD [10] and newly developed somatic symptoms were observed in patient with PTSD [11]. However, this relationship between PTSD and somatization has been inconsistent in sexually abused children due to a dearth of research. While an earlier study found that somatization was comorbid in patients with PTSD due to child sexual abuse [12], PTSD and somatization were not related in sexually abused women in a recent study [13]. Thus, it can be a new research topic to investigate the association between PTSD and somatization and other factors influencing the relationship between PTSD and somatization.

As one of the factors, the type of the trauma is assumed to associate with the post-traumatic response of the survivors. Sexually abused victims reported higher emotional reactions during and after trauma compared to victims with other trauma [14]. In terms of type of sexual abuse, survivors of abuse involving penetration exhibited higher prevalence rates of PTSD than survivors of non-contact assaults [15, 16]. In contrast, questions have been raised that this correlation trend can change in cases of extreme traumas. It has been suggested to reconsider the definition of trauma which is severe enough to cause PTSD because people actually suffer from PTSD due to sub-traumatic life events [17]. A study reported that penetrative sexual abuse was not significantly correlated with post-traumatic somatization while sexual abuse without penetration was [18]. Thus, it can be beneficial to investigate the influence of the type of sexual abuse on PTSD, somatization, and also on the association between the PTSD and somatization in sexually abused victims. It is also found that lower intelligence increases risk for PTSD [19] and functional somatic symptoms [20]. Although there have been findings that higher negative cognitions after sexual abuse were associated with somatizations [21] and the severity of PTSD in sexual assault victims [22], the relationship between the intelligence and post-traumatic somatization remain unclear in child sexual abuse.

Thus, we hypothesized that PTSD symptoms would correlate with somatization and type of sexual abuse would have an influence on the relationship between PTSD and somatization in victims of child sexual abuse. We also hypothesized that intelligence would be associated with post-traumatic somatization. The aims of the present study were i) to evaluate the relationship between somatizations and PTSD symptoms in child sexual abuse victims, ii) to determine whether type of abuse has a moderating effect on the effect of PTSD symptoms on somatization, and iii) to evaluate whether intelligence is associated with somatization.

## Methods and materials

### Participant enrollment

This study included 63 sexual abuse victims between 8 and 16 years of age who were recruited from individuals who visited the Sunflower Children’s Center in South Korea during a 2-year period. The Sunflower Children’s Centers are public centers for sexually abused children and adolescents that provide psychological counseling, medical consultation, and assistance with legal advice and criminal investigations. Of the 133 subjects who were recruited for the study, 46 were not included in the study because their data lacked in two or more items among the case characteristics, PTSD symptom, somatization, or intelligence and 24 did not agree to participate in the study; thus, 63 participants were assessed.

### Subject characteristics

All participants underwent individual interviews with a trained social worker, psychologist, or child psychiatrist to obtain demographic and other relevant data; caregivers were interviewed separately from the victims. Parents completed questionnaires about the somatization of the victims, and the victims were evaluated to determine their level of intelligence and degree of traumatic symptoms. Type of abuse was categorized as rape or sexual molestation: rape was defined as penetrative abuses (vaginal, anal, or oral penetration) of the victim or attempted penetration; and sexual molestation was defined as other than penetrative abuse involving sexual stimulation, such as the touching of body parts or indecent exposure.

### Measurements

The somatizations of the participants were evaluated with the Child Behavioral Checklist/6-18 (CBCL; [23]. This questionnaire is completed by parents to assess the behavioral and emotional problems and competence of a child during the preceding 6 months in terms of six problem items that are scored from 0 (none) to 2 (frequent): anxious/depressed, withdrawn, sleep problems, somatic problems, aggressive behavior, and destructive behavior. Additional competence items on the CBCL assess the child’s activities, social relations, and school functioning. The somatizations of the participants were evaluated using the somatic problem scores of the Korean version of CBCL [24]. The K-CBCL was included in a large multicultural study for its factor structure and due to its validation with the CBCL [25]; T-scores were calculated for each domain.

The post-traumatic symptoms of the participants were evaluated with the Trauma Symptom Checklist for Children (TSCC; [26], which was designed to assess symptoms related to sexual abuse and other traumas using six clinical scales: anxiety, depression, post-traumatic stress, sexual concerns, dissociation, and anger. TSCC is a self-report instrument and it does not provide a cut-off for a PTSD diagnosis. A validation study of the Korean version of the TSCC reported good levels of internal consistency, test–retest reliability, and concurrent and discriminative validity [27]. Each domain score is calculated as a T-score, and the post-traumatic stress subscale was used to evaluate the intensity of PTSD symptoms in the present study.

The intelligence level of each participant was evaluated with Korean version IV of the Wechsler Intelligence Scale for Children (K-WISC-IV; [28], which has been standardized and validated for the Korean population [29]. The K-WISC-IV includes assessments of full-scale IQ (FSIQ) and four indices of cognitive functioning: the verbal comprehension index, the perceptual reasoning index, the freedom from distractibility index, and the processing speed index. FSIQ and subscale scores were adjusted for age. The present study analyzed the FSIQ but did not group participants according to intellectual disability, borderline intellectual functioning, or normality.

All evaluations were conducted before the initiation of the therapies, such as sex education, play therapy, art therapy, psychotherapy, or pharmacotherapy if participant needed any kind of therapies. Written informed consent was obtained from all participants and their legal guardians, and this study was approved by the Institutional Review Board of Gachon University Gil Medical Center.

### Statistical analyses

Demographic data were analyzed with descriptive analysis. Kolmogorov–Smirnov test was used to test normal distribution of data. Correlations between PTSD symptoms and somatization were analyzed with Pearson’s correlation coefficients when the data were normally distributed and with Spearman’s correlation coefficients when they were not. A multivariate regression analysis was performed for somatization based on age, intelligence, PTSD symptoms, and type of abuse. The effect of the interaction between PTSD symptoms and type of abuse on somatization was analyzed in a multiple regression analysis using interaction terms. Additionally, the correlation between PTSD and somatization was evaluated for each type of abuse (rape and molestation) and with a multiple regression analysis. All analyses were performed with SPSS for Windows (SPSS, version 23; Chicago, IL, USA), and *p* values < 0.05 (two-way) were considered to indicate statistical significance.

## Results

### Demographic data and case characteristics

Data on the demographic and sexual abuse experienced by participants are presented in Table 1. The median age of the total sample was 11 years, 92% of participants were female, and the mean FSIQ was 90.63 ± 16.27. In total, 73% (*n* = 46) of the subjects reported abuse that met our criteria for molestation, and 27% (*n* = 17) reported abuse that met our criteria for rape. Most perpetrators were male (98%, *n* = 62) and acquaintances of the victim (79.4%, *n* = 50).

**Table 1:** Characteristics of study subjects.

Variables			Number (%)
Individual characteristics			63 (100)
	Sex		
		Male	5 (7.9)
		Female	58 (92.1)
	Age		11.00 [9.00–13.00] ^a^
	IQ		90.63 ± 16.27 ^b^
Caregivers			
	Both parents		31 (49.2)
	Single parent		25 (39.7)
	Relatives		5 (7.9)
	Institution		2 (3.2)
Perpetrator(s)			
	Male		62 (98.4)
	Female		1 (1.6)
	Single perpetrators		59 (93.7)
	Multiple perpetrators		4 (6.3)
Type of abuse			
	Molestation		46 (73.0)
	Rape		17 (27.0)
Victim–perpetrator relationship			
	Stranger		13 (20.6)
	Acquaintance		50 (79.4)
		Family member	19
		Other than family member	31

^a^ Data are shown as median [interquartile range]

^b^ Data are shown as mean (standard deviation)

### Effects of PTSD on somatization and other related factors

Table 2 shows the positive correlation between PTSD symptoms and somatization (*rho* = 0.372, *p* = 0.003) and negative correlation between FSIQ and age (*rho* = -0.397, *p* = 0.001). PTSD symptom (*p* = 0.078) and FSIQ (*p* = 0.200) showed normal distribution while age (*p* = 0.032) and somatization score (*p* < 0.001) did not. Table 3 shows the results of the multivariate regression analyses for somatization (log-transformed). In the final model (F = 3.330, *p* = 0.011, R^2^ = 0.234), somatization was positively associated with intelligence (β = 0.327, *p* = 0.021) and PTSD symptoms (β = 0.471, *p* = 0.001) but not with age (β = 0.102, *p* = 0.468) or type of abuse (β = 0.158, *p* = 0.281). However, the interaction between type of abuse and PTSD symptom was positively associated with somatization (type of abuse*PTSD symptom, β = -0.299, *p* = 0.047).

**Table 2:** Correlations among PTSD, somatization, age, and FSIQ.

		PTSD symptom	Somatization score	Age	FSIQ
	PTSD symptom	―	*rho* = 0.372 ^a^ *p* = 0.003	*rho* = 0.164 *p* = 0.211	*r* = -0.081 *p* = 0.540
	Somatization score		―	*rho* = 0.025 *p* = 0.844	*rho* = 0.121 *p* = 0.344
	Age			*―*	*rho* = -0.397 ^a^ *p* = 0.001
	FSIQ				*―*
Mean ± SD or Median [IQR]		47.80 ±12.80	58.00 [50.00–64.00]	11.00 [9.00–13.00]	90.63 ± 16.97

^a^ *p* < 0.05

Pearson’s correlation coefficient for normally distributed data and Spearman’s correlation coefficient for data which are not normally distributed.

PTSD symptoms: Post-traumatic Stress Disorder symptoms scores on the Trauma Symptom Checklist for Children (presented as T-score), Somatization score: somatic problem score on the Child Behavior Checklist (presented as T-score)

FSIQ: full scale intelligence quotient, SD: standard deviation, IQR: interquartile range

**Table 3:** Factors associated with somatization in sexually abused children and adolescents.

Factors	Model 1			Model 2			Model 3		
	B	β	*p* value	B	β	*p* value	B	β	*p* value
Age	0.013	0.194	0.172	0.007	0.106	0.466	0.007	0.102	0.468
Intelligence	0.002	0.238	0.095	0.002	0.251	0.071	0.003	0.327	0.021^a^
Type of abuse (Rape)				0.021	0.062	0.661	0.053	0.158	0.281
PTSD symptom				0.004	0.338	0.010^a^	0.006	0.471	0.001^b^
Type of abuse (Rape) * PTSD symptom							-0.008	-0.299	0.047^a^
Model fitness	F = 1.733, *p* = 0.186			F = 2.925, *p* = 0.029^a^			F = 3.300, *p* = 0.011^b^		

Multivariate linear regression analysis with somatization (log-transformed) as the dependent variable (predictors: age, intelligence, type of abuse, PTSD symptom, and type of abuse*PTSD symptom)

^a^ *p* < 0.05

^b^ *p* < 0.01

In addition, correlation and regression analyses were performed only in female victims (*n* = 58, Table A in S1 Appendix). PTSD symptom positively correlated with somatization (Table B in S1 Appendix; *r* = 0.353, *p* = 0.008) and PTSD had a moderating effect on somatization in the final model of multivariate linear regression analysis (Table C in S1 Appendix; β = 0.457, *p* = 0.003). The type of abuse was found to moderate the effect of PTSD symptom on somatization, with quite moderate significance in the regression analysis (β = -0.295, *p* = 0.065, R^2^ = 0.232).

### Influence of type of abuse on the effect of PTSD on somatization

**Fig 1:**
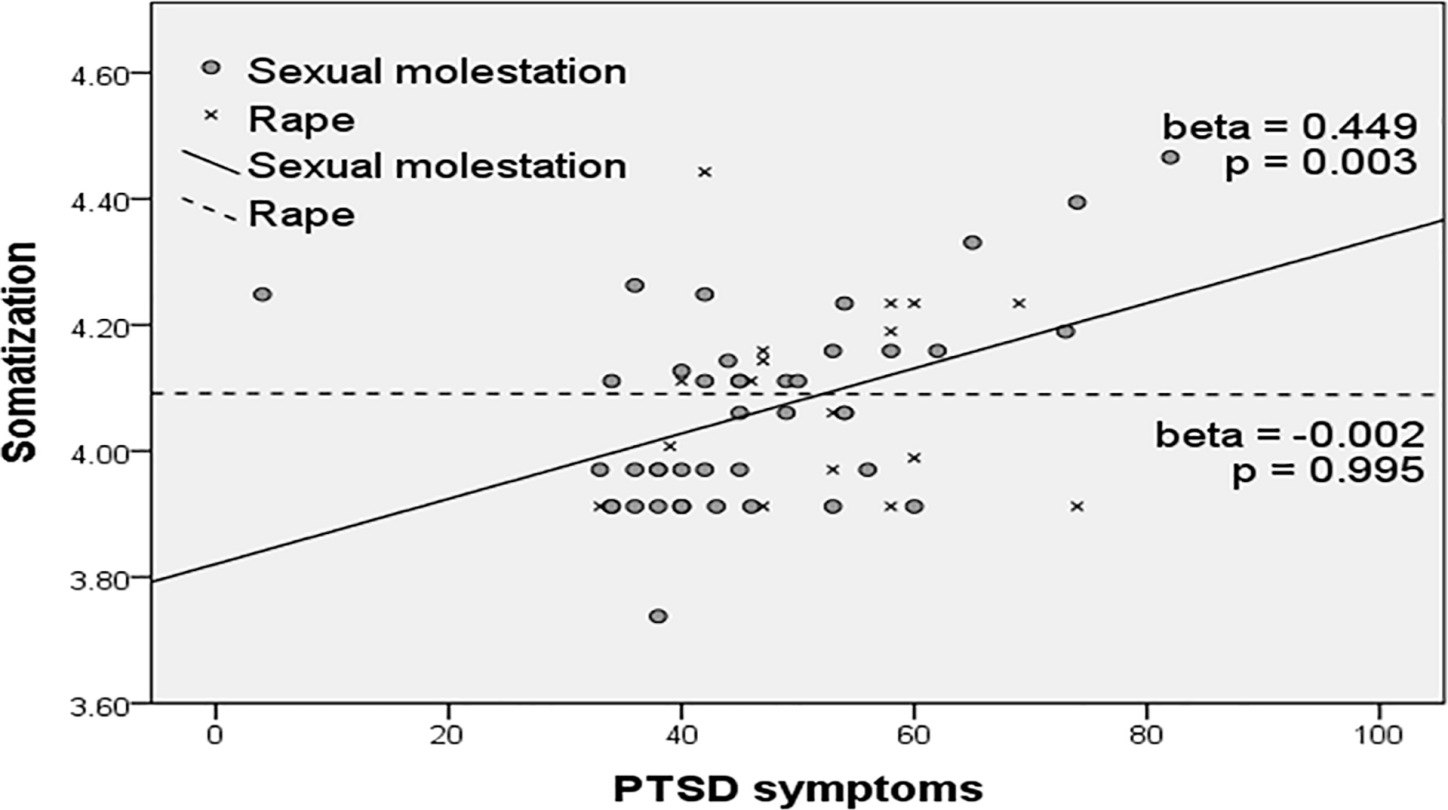
Moderating effect of type of abuse on the relationship between PTSD symptoms and somatization. PTSD: Post-traumatic Stress Disorder symptom score on Trauma Symptom Checklist for Children, Somatization: somatic problem score on Child Behavior Checklist (log-transformed).

Multivariate regression analyses were performed according to type of abuse (Fig 1). PTSD symptoms did not significantly predict somatization in the rape group (β = -0.002, *p* = 0.995) but were significantly correlated with somatization in the sexual molestation group (β = 0.449, *p* = 0.003).

## Discussion

In the present study, PTSD symptoms correlated with somatization in sexually abused children. More specifically, intelligence and PTSD symptoms were associated with somatization, whereas age and type of abuse were not. Type of abuse alone had no effect on somatization, but it had moderating influence on the effect of PTSD symptoms on somatization.

The present findings are in line with those of several previous studies. The negative correlation between intelligence and age was found in this study. Previous studies have shown that the risk of being sexually abused decreases with age for intellectually normal children, whereas the risk increases or maintained with age for intellectually disabled children [30, 31]. Our finding is in accordance with the earlier findings showing negative correlation between age and intelligence in sexually abused children and adolescents. The major finding of our study is the positive association between PTSD symptom and somatization, which is similar to the existing results. A prospective study of traumatized patients found that PTSD increases the risk of a somatization disorder relative to other psychiatric disorders [32] and, among depression, anxiety, and other demographic variables, PTSD is the best predictor of somatization in female patients with PTSD [33]. Additionally, the intensity of PTSD symptoms also has been found to correlate with somatization. Higher levels of somatic symptoms are associated with a higher frequency of post-traumatic symptoms in combat veterans with PTSD [34]. Although previous studies reported functional somatic symptoms such as fibromyalgia [35], gastroenterological symptoms [36], chronic pelvic pain [37], musculoskeletal symptoms [8] in sexually abused victims, only a few studies have investigated the relationship between PTSD and somatization in child sexual abuse victims [12, 13]. Because we used TSCC which comprehensively evaluates PTSD symptoms including sexual abuses and other traumas, it was possible to find that the intensity of PTSD symptom correlated with the severity of somatization in sexual abuse victims in the present study. We consider that this positive correlation between PTSD symptom and somatization may be explained by the functional consequences of PTSD itself, such as social and occupational dysfunction and physical disabilities. This relationship may also be explained by the contributions of arousal, dissociation, and comorbidity as well as the psychological assumption of lowered responsiveness to external stimuli in conjunction with an increased awareness of internal stimuli [38]. However, the possibility that somatization is related to sexual abuse itself still remained, and even if not, we cannot confirm the causality between the PTSD and somatization with this result.

Higher intelligence showed association with more severe somatization in child sexual abuse victims in the present study. Several prior studies found that individual with a lower level of intelligence exhibited higher somatization [20, 39] and patients with somatization also expressed more cognitive complaints, including deficits in psychomotor speed and attention [20]. In child sexual abuse victims, findings have been replicated that abused subjects showed impaired cognitive function compared to the control group [40, 41]. However, we found that higher intelligence is associated with highly reported somatic symptoms in this study. This finding can be attributed to our study sample comprised of child sexual abuse victims and the functional aspects of the somatization itself. High intelligence is one of the protective factor for psychiatric diagnosis in sexual abuse victims, but it can also act as a vulnerability factor [42]. Child sexual abuse victims with high intelligence may be more sensitive to their environments or themselves, which makes them more susceptible and use their variety of adaptive handling models compared to victims with low intelligence [42]. Thus, it is possible to assume that positive association between high intelligence and increased somatization in our study can be a result of the coping mechanism.

The present study also showed that type of abuse moderated the effect of PTSD symptoms on somatization in sexual abuse victims. Type of abuse itself was not correlated with somatization and had only a moderating influence on the effect of PTSD symptoms on somatization. Depending on the type of abuse, PTSD symptoms had an effect on somatization in the molestation group. However, this effect was not significant when analyzed in the rape group, which is the most severe type of sexual abuse. Regarding the concept of trauma, it has been suggested that people who develop PTSD symptoms after sub-traumatic stressors, such as divorce, unemployment, or chronic illness, would have different underlying psychobiological mechanisms than victims suffering from severe trauma, such as combat or rape [17, 43]. The present results are in line with these findings and further show that this mechanism occurs in child sexual abuse victims as well. Thus, in extreme cases of sexual abuse, predisposing factors likely play a secondary role [17]. The reasons the significant relationship between somatization and PTSD symptoms disappeared in cases of rape remain unclear. It is possible that because rape is an extremely severe form of trauma, even the positive and adaptive effects of somatization may not be effective for victims of rape. From a compensatory perspective, somatization may be an adaptive response to psychological distress. In cancer survivors with PTSD symptoms, somatization serves as an indicator of traumatic distress in patients with repressive adaptive coping styles and low levels of reported distress [9]. In patients with multiple functional somatic symptoms, somatization has been associated with high scores on the coping scale which, in turn, are correlated with poor verbal ability and executive functioning [20]. These authors suggested that poor verbal ability plays a role in increasing reliance on somatization as a coping strategy and may explain the relationship between intelligence and somatization in the present study. In this manner, the rape type of abuse neutralizes somatization and its adaptive functions regardless of PTSD symptoms, which are the strongest predictor of somatization.

The present study has several limitations that should be noted. Although the TSCC identifies post-traumatic symptoms reasonably well, our omission of a formal diagnostic process in which a psychiatrist determined whether subjects met criteria for PTSD limits the interpretation of the mechanism described in this study. Additionally, the relatively small sample size may affect the generalizability of the present results, and the lack of demographic information and detailed case characteristics such as emotional neglect, violence during sexual abuse, number and frequency of trauma, and disclosure pattern in the analyses may also limit the findings. The classification of the type of sexual abuse is vague because raped participants can be also sexually molested. The sex effect was not considered in this study and it should be considered in future studies with larger samples because the moderating effect of the type of abuse on the influence of PTSD symptom on somatization decreased in only-female victims in our result. This will elucidate the sex difference of the response mechanism after sexual abuse regarding the case characteristics, PTSD, and somatization. In the context of quantifying the trauma severity, investigations on other measures such as number of perpetrators, time of onset, or frequency of the trauma also could have generalized the results.

Despite these shortcomings, the present study found that intelligence and PTSD symptoms are associated with somatization in child sexual abuse victims and showed that the rape type of abuse may neutralize the predictive effects of PTSD regarding somatization. Additionally, the type of abuse had precedence over PTSD symptoms in terms of predicting somatization in sexual abuse victims. Thus, extreme care must be taken when assessing victims of rape because they may be so incapacitated that they can fail to report somatic complaints or psychological distress. Further research investigating the psychobiological mechanisms underlying post-traumatic somatization in child sexual abuse victims will be necessary.

## Supporting information

S1 Appendix Table A. Characteristics of study subjects (female subjects only).
Table B. Correlations among PTSD, somatization, age, and FSIQ (female subjects only).
Table C. Factors associated with somatization in sexually abused children and adolescents (female subjects only).
(DOC)

S2 Appendix (XLSX)
