# Supplementary material for: PTSD correlates with somatization in sexually abused children: Type of abuse moderates the effect of PTSD on somatization
Source: PLoS One. 2018 Jun 21;13(6):e0199138. doi: 10.1371/journal.pone.0199138 (PMC6013160; doi:10.1371/journal.pone.0199138)
Supplement: S1 Appendix — Table A. Characteristics of study subjects (female subjects only). Table B. Correlations among PTSD, somatization, age, and FSIQ (female subjects only). Table C. Factors associated with somatization in sexually abused children and adolescents (female subjects only). (DOC) [file pone.0199138.s001.doc]

**Table A.** Characteristics of study subjects (female subjects only)

| Variables | | | | Number (%) |
| --- | --- | --- | --- | --- |
| Individual characteristics | | | | 58 (100) |
|  | | Sex | |  |
|  | |  | Male | 0 (0) |
|  | |  | Female | 58 (100) |
|  | | Age | | 11.03 ± 2.30 ^a^ |
|  | | IQ | | 91.33 ± 16.95 ^a^ |
| Caregivers | | | |  |
|  | | Both parents | | 28 (48.3) |
|  | | Single parent | | 23 (39.7) |
|  | | Relatives | | 5 (8.6) |
|  | | Institution | | 2 (3.4) |
| Perpetrator(s) | | | |  |
|  | | Male | | 54 (93.1) |
|  | | Multiple perpetrators | | 4 (6.9) |
| Type of abuse | | | |  |
|  | Molestation | | | 43 (74.1) |
|  | Rape | | | 15 (25.9) |
| Victim–perpetrator relationship | | | |  |
|  | | Stranger | | 11 (19.0) |
|  | | Acquaintance | | 47 (81.0) |
|  | |  | Family member | 18 |
|  | |  | Other than family member | 29 |

^a^ Data are shown as mean ± standard deviation

**Table B.** Correlations among PTSD, somatization, age, and FSIQ (female subjects only)

|  | | PTSD symptom | Somatization score | Age | FSIQ |
| --- | --- | --- | --- | --- | --- |
|  | PTSD symptom | ― | *rho* = 0.355 ^a^  *p* = 0.008 | *rho* = 0.158  *p* = 0.248 | *r* = -0.067  *p* = 0.625 |
|  | Somatization score |  | ― | *rho* = 0.060  *p* = 0.652 | *rho* = 0.131  *p* = 0.328 |
|  | Age |  |  | *―* | *rho* = -0.379 ^a^  *p* = 0.003 |
|  | FSIQ |  |  |  | *―* |
| Mean ± SD | | 48.13 ±13.20 | 58.00  [50.00-64.00] | 11.00  [9.00-13.00] | 91.33 ± 16.95 |

^a^ *p* < 0.05

Pearson’s correlation coefficient for normally distributed data and Spearman’s correlation coefficient for data which are not normally distributed.

PTSD symptoms: Post-traumatic Stress Disorder symptoms scores on the Trauma Symptom Checklist for Children (presented as T-score), Somatization score: somatic problem score on the Child Behavior Checklist (presented as T-score)

SD: standard deviation, FSIQ: full scale intelligence quotient

**Table C.** Factors associated with somatization in sexually abused children and adolescents (female subjects only)

| Factors | Model 1 | | |  | Model 2 | | |  | Model 3 | | |
| --- | --- | --- | --- | --- | --- | --- | --- | --- | --- | --- | --- |
|  | B | β | *p* value |  | B | β | *p* value |  | B | β | *p* value |
| Age | 0.015 | 0.219 | 0.133 |  | 0.010 | 0.147 | 0.328 |  | 0.009 | 0.134 | 0.362 |
| Intelligence | 0.002 | 0.252 | 0.085 |  | 0.002 | 0.257 | 0.074 |  | 0.003 | 0.337 | 0.023^a^ |
| Type of abuse (Rape) |  |  |  |  | 0.009 | 0.027 | 0.853 |  | 0.045 | 0.129 | 0.402 |
| PTSD symptom |  |  |  |  | 0.004 | 0.326 | 0.017^a^ |  | 0.005 | 0.457 | 0.003^b^ |
| Type of abuse (Rape)  * PTSD symptom |  |  |  |  |  |  |  |  | -0.007 | -0.295 | 0.065 |
| Model fitness | F = 2.000, *p* = 0.146 | | |  | F = 2.672, *p* = 0.043^a^ | | |  | F = 2.959, *p* = 0.021^a^ | | |

Multivariate linear regression analysis with somatization (log-transformed) as the dependent variable (predictors: age, intelligence, type of abuse, PTSD symptom, and type of abuse*PTSD symptom)

^a^ *p* < 0.05, ^b^ *p* < 0.01
